# Environmental Regulation Using *Plasticoding* for the Evolution of Robots

**DOI:** 10.3389/frobt.2020.00107

**Published:** 2020-10-01

**Authors:** Karine Miras, Eliseo Ferrante, A. E. Eiben

**Affiliations:** ^1^Computer Science Department, Vrije Universiteit Amsterdam, Amsterdam, Netherlands; ^2^Autonomous Robotics Research Centre, Technology Innovation Institute, Abu Dhabi, United Arab Emirates

**Keywords:** evolutionary robotics, morphological evolution, phenotypic plasticity, environmental regulation, locomotion, environmental effects

## Abstract

Evolutionary robot systems are usually affected by the properties of the environment indirectly through selection. In this paper, we present and investigate a system where the environment also has a direct effect—through regulation. We propose a novel robot encoding method where a genotype encodes multiple possible phenotypes, and the incarnation of a robot depends on the environmental conditions taking place in a determined moment of its life. This means that the morphology, controller, and behavior of a robot can change according to the environment. Importantly, this process of development can happen at any moment of a robot's lifetime, according to its experienced environmental stimuli. We provide an empirical proof-of-concept, and the analysis of the experimental results shows that environmental regulation improves adaptation (task performance) while leading to different evolved morphologies, controllers, and behavior.

## 1. Introduction

What makes natural life remarkably complex goes beyond having genes encoding a trait or behavior, as it also concerns mechanisms in the DNA that regulate the expression of these genes as a function of environmental conditions. That is, genes should be activated “at the right place at the right time.” An amazing number of 95% of DNA does not code for any protein: Part of it is responsible for regulation^1^ (Sapolsky, 2017). In fact, the more genomically complex an organism is, the larger the percentage of the DNA that is devoted to environmental regulation (Sapolsky, 2017). This regulation happens through a process once called *epigenetics* (Bossdorf et al., 2008), a term that recently has been utilized only in cases when this regulation results in heritable regulatory changes (Sapolsky, 2017). One of the results of this regulation is lifetime *phenotypic plasticity*, and it concerns the capacity of an individual to develop aspects of its phenotype, such as morphology, physiology, synaptic connections, in response to given environmental stimuli during its lifetime (Fusco and Minelli, 2010).

Although phenotypic changes like learning (Fusco and Minelli, 2010) and training (Kelly et al., 2011) are also examples of *phenotypic plasticity*, here we consider only phenotypic changes that happen through regulation. *Phenotypic plasticity*is pervasive in nature and may accelerate, decelerate, or have an insignificant effect on evolutionary change (Price et al., 2003). Some examples of lifetime *phenotypic plasticity* acting on the body are Passerine birds that change their musculature to cope with winter (Liknes and Swanson, 2011), and several vertebrate species that suffer color changes in different seasons (Mills et al., 2018). As for behavioral changes caused by environmental regulation acting on the brain, think of the physiology of a mother changing to produce milk when she smells her baby—this is a change that happens through the activation of genes. More specifically, this example is thoroughly described by Sapolsky (2017) as “A female smells her newborn, meaning that odorant molecules that floated off the baby bind to receptors in her nose. The receptors activate and (many steps later in the hypothalamus) a transcription factor activates, leading to the production of more oxytocin. Once secreted, the oxytocin causes milk letdown. Genes are not the deterministic holy grail if they can be regulated by the smell of a baby's posterior. Genes are regulated by all the incarnations of environment. Promoters and transcription factor introduce *if/then clauses*: *If* you smell your baby, *then* activate the oxytocin gene.”

Within engineering, the research areas related to artificial evolution are that of Evolutionary Computing (Eiben and Smith, 2003, 2015) and Evolutionary Robotics (Nolfi and Floreano, 2000; Doncieux et al., 2015; Nolfi et al., 2016). These fields have addressed the evolution of robot controllers (brains) with considerable success but evolving the morphologies (bodies) has received much less attention (Prabhu et al., 2018). Importantly, the influence of the environment has been even more scarcely investigated. Although it is not uncommon to use developmental encodings: GRNs (Bongard, 2002) simulate development with local interactions; CPPNs propose an abstraction to development without local interaction (Stanley, 2007); an approach inspired on Hox Genes (Samuelsen et al., 2013). However, there is no substantial work in the literature that successfully allows a genotypic structure to be regulated by changes in external environmental conditions. The key idea of this paper is to develop a novel robot DNA structure, that is, a new encoding method that endows robots with lifetime *phenotypic plasticity*, and with it, demonstrate the benefits of environmental regulation. We achieve this through a genotype-phenotype mapping that responds and is modified according to the environmental conditions at given moments of a robot's life. This idea represents a significant departure from existing systems, where the genotype-phenotype mapping is “injective,” that is, each genotype encodes only one possible phenotype. This holds true for both direct and indirect (e.g., generative or developmental) mappings (Rothlauf, 2006). In contrast, here we study genotype-phenotype mappings where a genotype encodes multiple possible phenotypes and the actual “incarnation” (the robot body and brain) depends directly on the environment external to the robot.

The expected benefits of *phenotypic plasticity* include higher efficiency and efficacy of robot evolution, together with increased responsiveness to environmental changes. We expect increased efficiency (speed) because an informed genotype-phenotype mapping makes reproduction less blind. Hence, the total number of trials (new robots born over the course of evolution) to evolve good robots should be lower than in systems using the conventional representations. Efficacy is the other side of the same coin given a fixed search budget (maximum number of trials for evolution), and an informed genotype-phenotype mapping will expectedly achieve better solutions. Last, but not least, a robot population that is equipped with an environment dependent genotype-phenotype mapping can cope with environmental changes better than a system where adaptation is induced through selection only. Importantly, by environmental changes, we refer not only to seasonal or permanent changes but also to the possibility of the same robot being able to deal with different environmental conditions as it moves about, e.g., from water to land, from flat land to a hill, etc.

While learning methods (Moshaiov and Abramovich, 2014; Miras et al., 2020a) could be applied to dealing with environmental changes, our *phenotypic plasticity* approach presents three main advantages. First, we allow changes not only to the brain but also to the body. While Evolutionary Robotics systems often focus on the evolution of the brain (Weigmann, 2012; Prabhu et al., 2018), we not only also permit the evolution of the body, but even its development. This is a fundamental refinement, given the known importance the body has for intelligence (Pfeifer and Bongard, 2006). Second, *phenotypic plasticity* allows an immediate change in response to environmental changes, as opposed to adaptation after multiple iterations. Finally, development does not have to deal with catastrophic forgetting. We believe that in the long term, ideally evolutionary robot systems should combine the benefits of evolution, development, and learning, as we observe in nature.

The specific objectives of this paper are:

1. To design a novel robot encoding with the capacity of *phenotypic plasticity* through environmental regulation during the robot lifetime. We call this robot encoding *Plasticoding*.
2. Use this robot encoding to demonstrate the benefits of environmental regulation to adaption when delaing with multiple environmental conditions, comparing this to a baseline robot encoding.

Additionally, we investigate this improvement in adaptation (performance on the task) by answering the following research questions:

1. What is the effect of *phenotypic plasticity* on the *morphological* properties?
2. What is the effect of *phenotypic plasticity* on the *controller* properties?
3. What is the effect of *phenotypic plasticity* on the emergent *behavior*?

## 2. Related Work

Existing work related to the evolution of virtual creatures dates back to the 1990s, when morphological (additionally to controller) evolution was addressed by Sims (1994). Sim's work was later put on a more solid footing by Pfeifer and Iida (2005).

In Bongard (2011) it has been shown that ontogenetic, i.e., lifetime development, can not only accelerate the discovery of successful behavior, but also produce robots that are more robust to variations of environmental conditions. Auerbach and Bongard (2014) utilized an information-theoretic measure of complexity to assess virtual creatures that evolved in a vast range of environments. The authors demonstrated that increasing the complexity of the environmental conditions might result in an increase in the morphological complexity of the creatures.

A developmental mechanism, presented as epigenetics, was proposed in Brawer et al. (2017), however, it was not dependent on environmental influences. The effect of different developmental mechanisms was studied in Kriegman et al. (2018b) by changing the stiffness of soft robots according to environmental changes. However, no improvement to evolvability was achieved by this. A similar investigation was presented in Kriegman et al. (2018a), this time obtaining improvements in evolvability. Nevertheless, although both these studies concern lifetime development mechanisms, the regulatory environmental changes were caused by the displacement of the robot itself, and therefore no actual “changing” environmental conditions were considered while robots always evolved in a flat plane. In Daudelin et al. (2018) reconfigurable robots evolved to cope with actual changes in the environmental conditions as they moved about, but no quantification of this effect on the morphological level was provided.

Finally, Risi and Stanley (2012) proposed a method for neural plasticity through which synapses can be modulated according to environmental conditions. This method differs from ours regarding the neural plasticity, in the sense that the plasticity happens directly in the phenotype, and not through the regulation of genetic material. Despite this difference in their mechanisms, in practice both methods result in immediate neural plasticity. Nevertheless, our work on environmental regulation has a long term view of being extended into epigenetics, i.e., patterns of gene expression resulting in plasticity could be temporarily inheritable, as opposed to acting directly (and only) on the phenotype of an individual. The inheritance of lifetime learning, which does not happen in nature, is common in neuroevolution and is referred to as Lamarckian evolution (Jelisavcic et al., 2017b). Still, it usually concerns inheritance of new neural connectivity patterns that are formed through life, whereas phenotypic changes caused by epigenetic modifications are not due to changes in genotype sequences, but only their expression. This way, while Lamarckian evolution causes cumulative “persistent” changes, epigenetic changes can be reversed after multiple generations (Slatkin, 2009). Additionally, through regulation we allow the conjoint plasticity of neural structure and body structure, as opposed to neural only. Notably, because plasticity has emerged in different complementing flavors in nature, it might be important to also combine them in artificial life. For example, in the future it could be interesting to combine regulation with neuro plasticity that takes place directly in the brain.

## 3. Methods

In our methodology, we use modular robots to represent the morphology (see section 3.1) and neural networks to represent the controllers (section 3.2). Together, these two represent the phenotypes, as they express the traits that ultimately, through the interaction with the environment, determine fitness. The evolutionary process acts on a higher level, the level of the genotypes, whose representation is explained in section 3.3.

**Figure 1:**
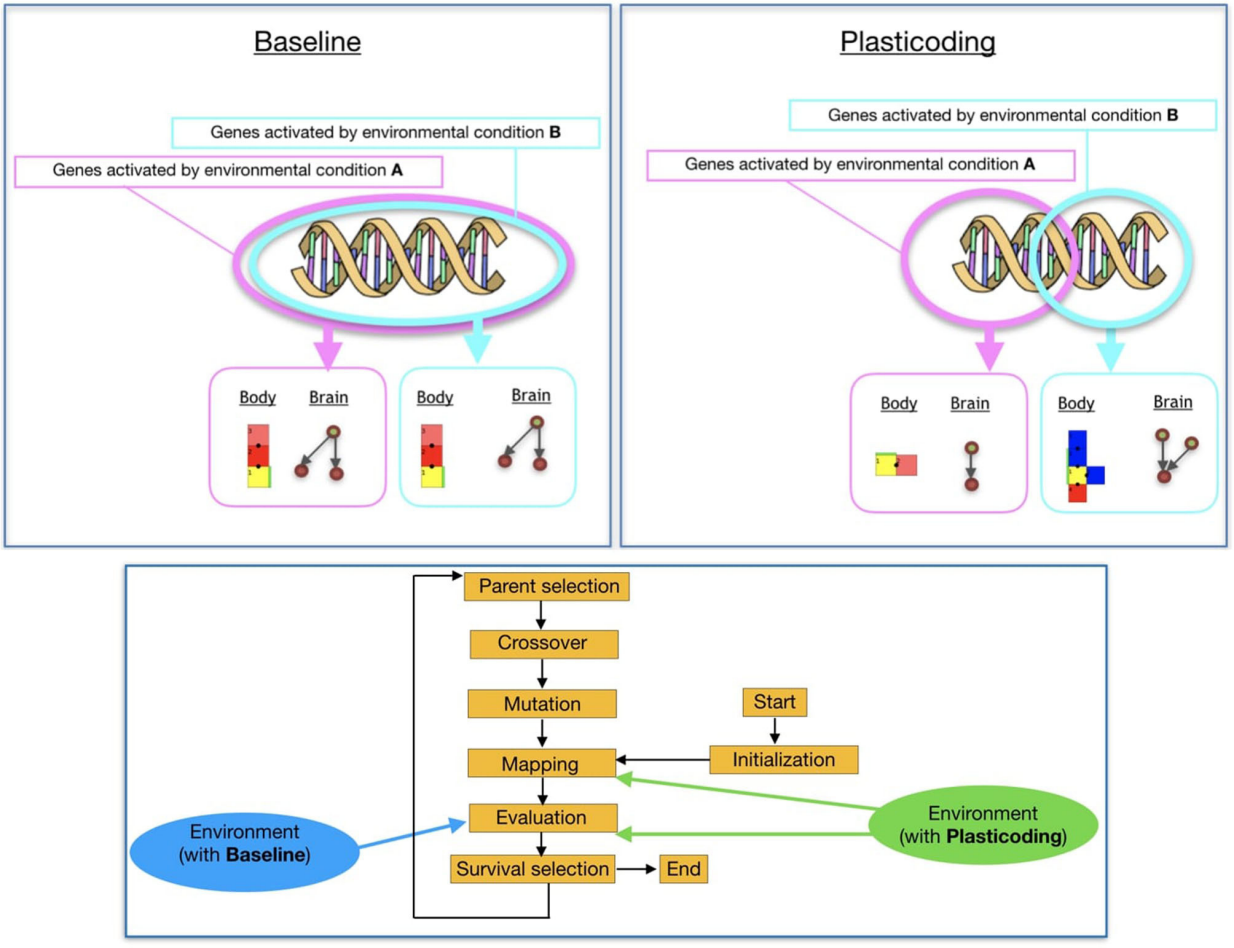
On the left, the robot modules: Core-component, which carries a controller electronic board and inertial measurement unit (IMU) sensors (C); Structural brick (B); Active hinges with servo motor joints in the vertical (A1) and horizontal (A2) axes; and Touch sensor (T). The polygons above the module's pictures are used to illustrate robot parts in the results section, and the letters are used to represent the modules in the robot representation. Modules C and B have attachment slots on their four lateral faces, and A1 and A2 have slots on their two opposite lateral faces; T has a single slot which can be attached to any slot of C or B. In the middle, an example of a robot (top-down view) before the simulation starts, while on the right, the same robot is shown during simulation.

In this paper, we extend a robot encoding we proposed in previous work (Miras et al., 2020b). Here, we refer to the previous encoding as *Baseline*, and to the new encoding as *Plasticoding*. The differential added by the *Plasticoding* concerns environmental regulation, allowing an individual to develop a different phenotype, i.e., morphology and/or controller, according to the conditions of the environment it is in (Figure 1, top). While for the *Baseline* the environment acts on the stage of the evaluation of the robots, for the *Plasticoding* it also acts on the stage of mapping the genotype to the phenotype (Figure 1, bottom). The methodology used for the regulatory mechanism is explained in section 3.4.

Genotypes are converted into phenotypes through a mapping process, which is explained in section 3.5. In the first generation, the genotype of the initial population is initialized according to the procedure described in section 3.6. During the evolutionary process, the operators of crossover and mutation are applied, which are explained, respectively, in sections 3.7 and 3.8. The overall evolutionary process is explained in section 3.9.

### 3.1. Robot Morphology

**Figure 2:**
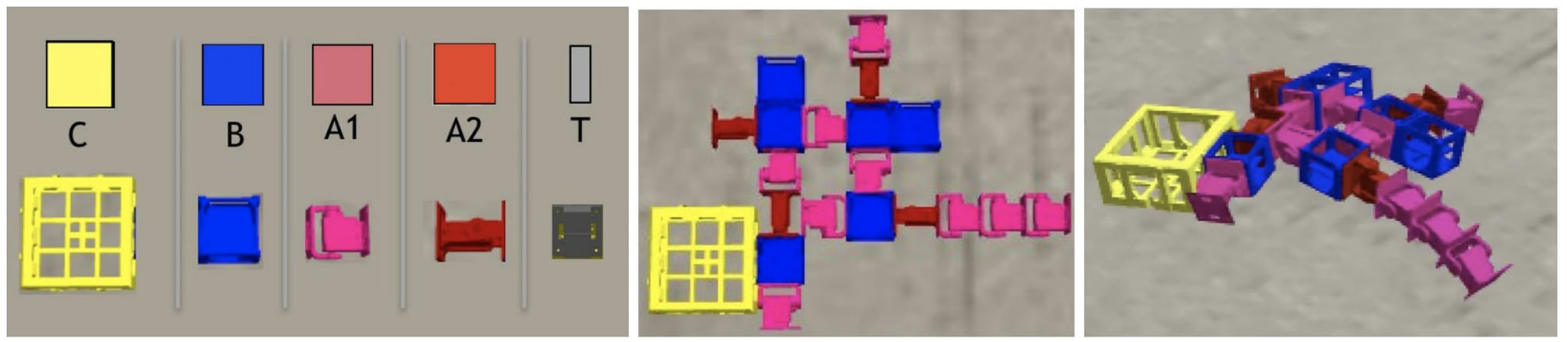
Example of controller: has a single oscillator neuron (with a recurrent connection), and a single input (sensor).

Each morphology phenotype (a “body”) is composed of modules (Auerbach et al., 2014) as shown in Figure 2, and the shape of the morphology is determined by evolution. Each module has a cuboid shape and has slots where other modules can attach. The morphologies can only develop in two dimensions, that is, the modules do not allow attachment to the top or bottom slots, but only to the lateral ones. There are five different types of modules, as reported in Table 1: core components, bricks, vertical joints, horizontal joints, and touch sensors. Any module can be attached to any module through its slots, except for the touch sensors, which cannot be attached to joints. Each module type is represented by a distinct symbol (see Table 1), and this is also the same language used in the genotype representation, described in section 3.3.

**Table 1:**
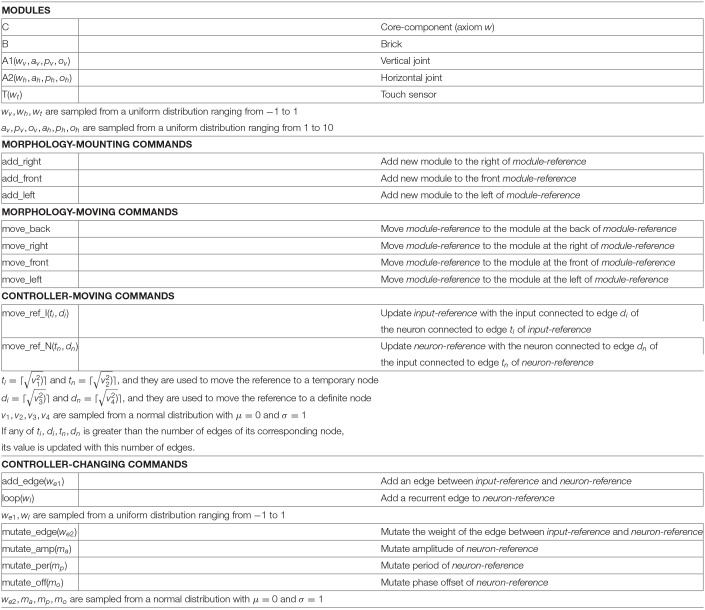
Alphabet of the grammars.

*Terminology is explained in section 3.5.3*.

Previous work (Jelisavcic et al., 2017a) demonstrated that this modular robot system functions in real hardware. For this, each module can be 3D printed, while the assembling of the modules and electronic parts (servos, sensors, board, etc.) is made manually. Production tutorials can be found in the link http://robogen.org/docs/video-tutorials.

### 3.2. Robot Controller

**Figure 3:**
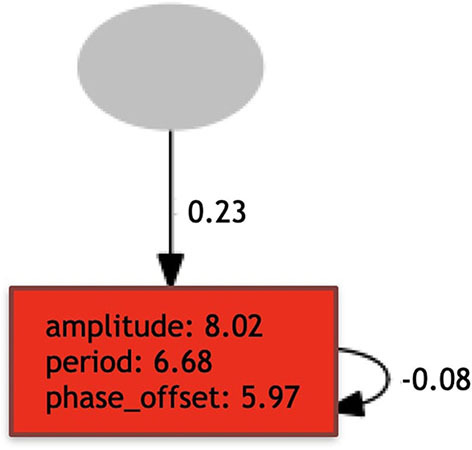
(Top) Effects of environmental regulation on phenotype. (Bottom) Effect of the environment on the evolutionary process of the population.

A controller phenotype (a “brain”) is a hybrid artificial neural network (Figure 3), which we call Recurrent Central Pattern Generator Perceptron (Miras and Eiben, 2019a). With hybrid we mean that we combine concepts from (a) CPGs, by having oscillator neurons; (b) Perceptrons, by having inputs connected to a single layer of neurons; (c) Recurrent neural networks, by allowing these neurons to have recurrent connections. In practice, the oscillator neurons generate a constant pattern of movement, and the sensor inputs can be used either to reduce or to reinforce movements, while the influence of these inputs can be remembered from each previous oscillation cycle.

Every aspect of the network is defined by evolution, and the network is formed by two types of nodes: input nodes associated with the sensor modules; and oscillator neuron nodes associated with the joint modules. For every joint in the morphology, there exists a corresponding oscillator neuron in the network, whose activation function is defined by Equation (1), which represents a sine wave defined by amplitude, period, and phase offset parameters. This activation function adjusts the output to fit the range of our servo motors, as proposed in Hupkes et al. (2018).

(1) $$\begin{array}{l} O=0.5-\frac{a}{2}+\frac{\text{sin}\left(\frac{2*\pi }{p}*(t-p*o))\right)+1}{2}*a \end{array}$$

where, *t* is the time step, *a* is the amplitude, *p* is the period, and *o* is the phase offset. The parameters *a*, *p*, and *o* can vary from 0 to 10. The different oscillator neurons cannot be directly interconnected, and every oscillator neuron may or may not possess a direct recurrent connection.

Additionally, for every sensor in the morphology, there exists a corresponding input in the network, and each input might connect to one or more oscillator neurons.

### 3.3. Genotype Representation

Our robot genotype is a generative model and is represented with an L-System inspired in Hornby and Pollack (2001), conjointly encoding both morphology and controller. L-Systems are parallel rewriting systems (Jacob, 1994) composed by a grammar defined as a tuple *G* = (*V, w, P*), where

- *V*, the alphabet, is a set of symbols containing replaceable and non-replaceable symbols.
- *w*, the axiom, is a symbol from which the generative process starts.
- *R* is a set of regulatory tuples (*c*, *p*) for the replaceable symbols, where *c* is a regulation clause and *p* is a production-rule.

Each genotype has distinct grammar, making use of the same alphabet (Table 1), and the alphabet is formed by symbols that represent types of morphological modules as well as commands for assembling modules and others for defining the structure of the controller. The symbols in the category Modules are replaceable, while the symbols of all other categories are non-replaceable.

### 3.4. Regulatory Clauses

We refer to information sensed from the environment by the robot as “environmental variables.” In the current experiments we utilize only one environmental variable, which describes the inclination of the ground in the environment and is represented by a term called *inclined*. The *inclined* environmental variable can be sensed by the IMU sensors of the robots, which provides data about the orientation of the robot in space. If the robot's center of mass has zero inclination, the term *inclined* assumes the value *False*, otherwise it assumes the value *True*. We utilized a single environmental variable because we have only two environments, which can be fundamentally differentiated by this single environmental variable. Notwithstanding, our methodology proposes that if needed, extra environmental variables can be derived from any robot sensors available, or even from data sources the robot might have at its reach, e.g., communication with other systems or robots.

Sapolsky (2017) coins a metaphor for the environmental regulation, calling regulation factors “*if then clauses*.” Here, we abstract this metaphor, and implement it in a literal sense. This way, for us a regulation clause is a Boolean expression, which is denoted by *c* in the tuple (*c*, *p*), while *p* is a production-rule. In *Plasticoding*, each clause contains up to *u* terms (in our case *u* = 2), and one same term may be repeated in the clause. Each term represents a comparison between an environmental variable and a value that can be *True* or *False*. The *u* terms are combined using *and* and *or* operators. Additionally, in *Plasticoding* every replaceable symbol from *V* appears in exactly *l* tuples (in our experiments we limited the study *l* = 2). This means that for each replaceable symbol, there are *l* = 2 pairs of the clause and production-rule, and the selection of the production-rules to be used for a replaceable symbol during development depends on the activation resulting from the regulatory clauses.

In contrast, *Baseline* is a special case: because there is no regulation, the clause *c* is always *True* and consequentially every replaceable symbol in *V* can only appear in one of the tuples, and therefore, has only one production-rule associated to it.

A few didactic examples of regulatory clauses are listed below:

**Ex.1:** *if inclined = True then …*

**Ex.2:** *if inclined = True or inclined = False then …*

**Ex.3:** *if inclined = False and hot = True then …* ^2^

Because we had only two environmental conditions in our experiments, we did not need more than two tuples, nor did we need more than two terms per clause. Nevertheless, when having many more environmental conditions to deal with, more genetic material will be necessary. This can be done by increasing the values of two parameters of *Plasticoding*: number of tuples per non-replaceable symbol *l*; maximum number of terms per clause *u*.

### 3.5. Genotype-Phenotype Mapping

For the *Baseline* method, the mapping from genotype to phenotype (the development), plays out in two stages that we call, respectively, *early* and *late* development. For the *Plasticoding*, the mapping plays out in three stages, with the regulation stage preceding the *early* development stage.

#### 3.5.1. Environmental Regulation

**Figure 4:**
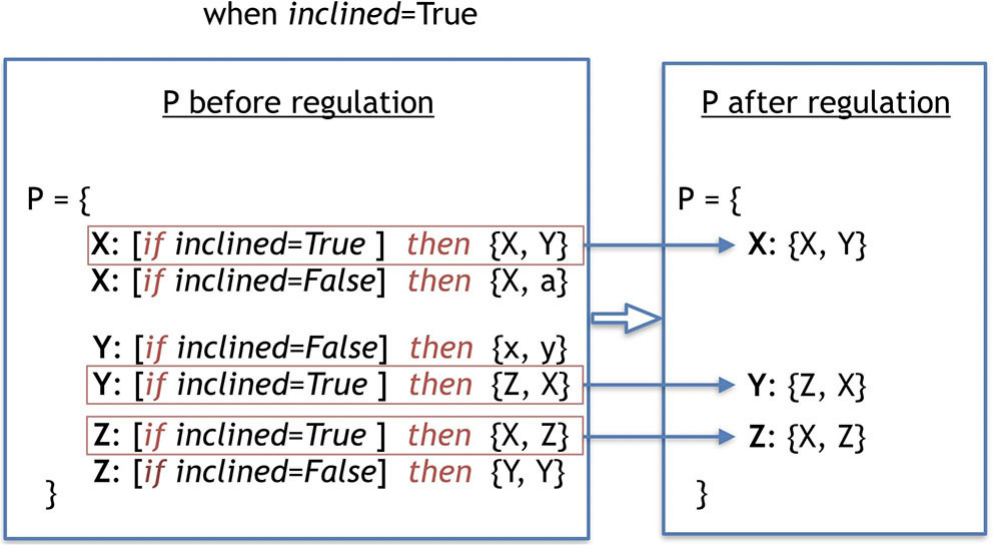
This example shows the process of regulation of a genotype, selecting its rules to be activated due to the environmental variable. The term used to define the environmental variable is called *included* and was sensed to be *True* when the regulation process took place. The production-rules in the second frame are the ones that became true given the environmental variable, and thus were selected to be in the final set *P*, which will be utilized in the *early* development.

The environmental regulation stage is responsible for selecting the production-rules that should be active during the *early* development stage, according to the environmental variables sensed by the robot. In the case of the *Baseline*, all production-rules are always active, because there is no regulation. Therefore, effectively, this stage does not occur at all in *Baseline*. In the case of the *Plasticoding*, for a production-rule to be active, its regulation clause must be *True*. Because multiple regulatory clauses for one replaceable symbol can be *True*, it is possible that multiple production-rules are activated. In this case, the multiple production-rules are concatenated sequentially as a single production-rule. Conversely, once multiple regulatory clauses can be *False*, it is possible that no production-rule gets activated. In this case the replaceable symbol is not replaced. Figure 4 depicts an example of a process of regulation of a genotype.

#### 3.5.2. Mapping Stage 1: Early-Development

The axiom *w* of the grammar is rewritten into a more complex string of symbols according to the activated production-rules of the grammar. During the rewriting, for a number of iterations *k* = 3, each replaceable symbol is simultaneously replaced by the symbols of its active production-rules. The following didactic example depicts the process of rewriting of our L-System representing one possible genotype, i.e., grammar. For the case of the *Plasticoding* method, it is assumed in this example that the regulation which activates production-rules has already been carried out.


W = X
V = {X, Y, Z, a}
P = {
X: {X,Y},
Y: {Z, a},
Z: {X, Z}
}


Given the above grammar, the rewriting is as follows:


Iteration 0: X
Iteration 1: X Y
Iteration 2: X Y Z a
Iteration 3: X Y Z a X Z a


The final string will contain non-replaceable symbols (Modules) and replaceable symbols (everything else). All these symbols can be interpreted with the process described hereafter.

#### 3.5.3. Mapping Stage 2: Late-Development

The early-developed phenotype from stage 1 is an intermediate phenotype made as a string of symbols, which must be mapped (late-developed) into a final phenotype. To aid the process of construction of the late-developed phenotype, multiple positional references (turtles) are kept: (a) a reference to the current module in the morphology, that we call a *module-reference*; (b) a reference to the current oscillator neuron of the neural network of the controller, that we call a *neuron-reference*; (c) a reference to the current sensor input of the neural network of the controller, that we call an *input-reference*; a reference to which the slot of the current module a new module should be attached to, that we call a *slot-reference*.

**Figure 5:**
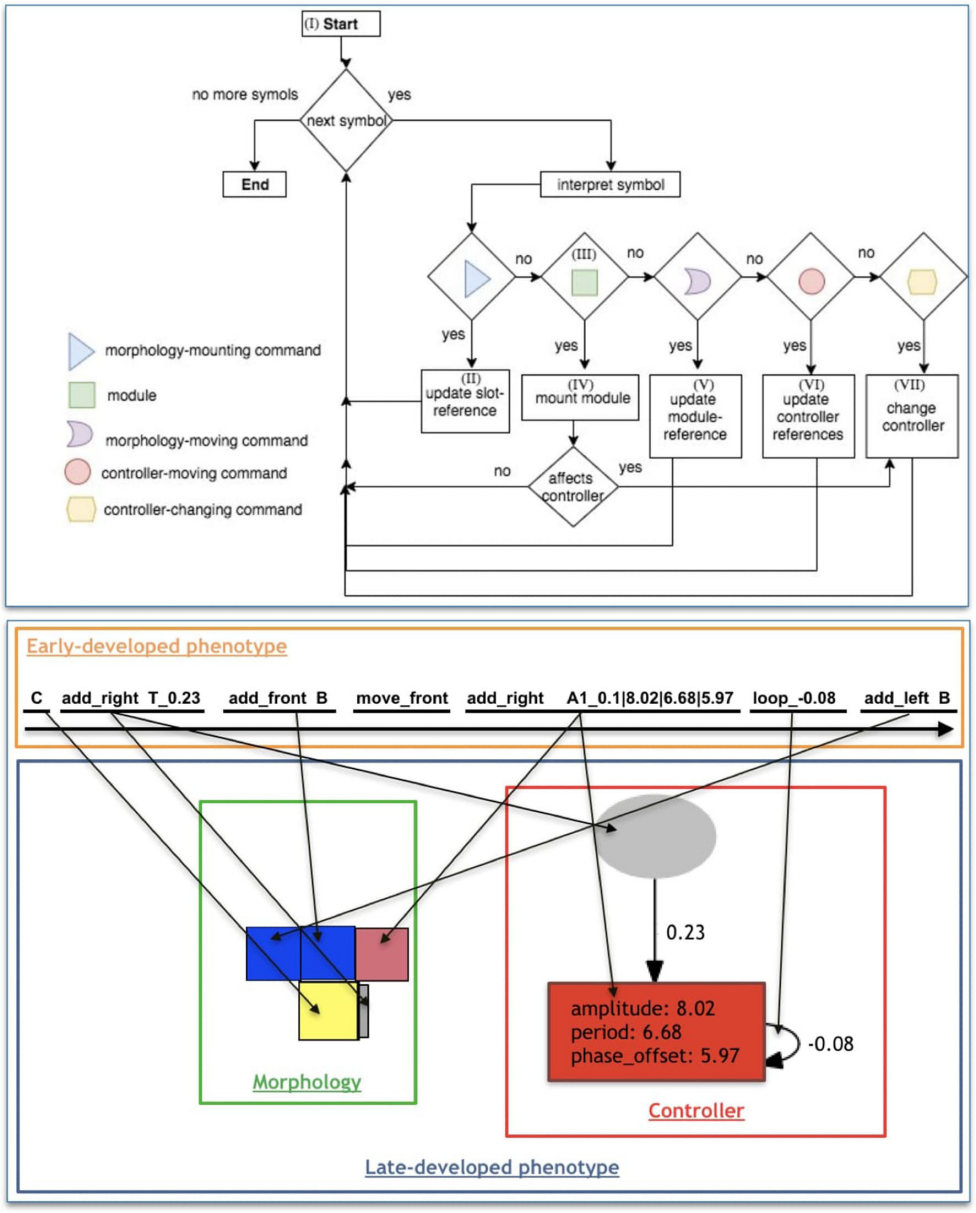
(Top) Flow-chart of the late-development process. From the left to right of the string, each symbol of the early-developed phenotype (string) goes through this process, being interpreted and developed (or not expressed). (Bottom) Illustration of decoding an early-developed phenotype into a late-developed phenotype with morphology and controller. From the left to right of the string, symbols are interpreted and developed, making incremental changes to the phenotype. An arrow going from the genotype to the phenotype should be interpreted as the process leading to the creation of the phenotype component pointed at by the arrow after the interpretation of the genotype component at the starting end of the arrow.

**Figure 6:**
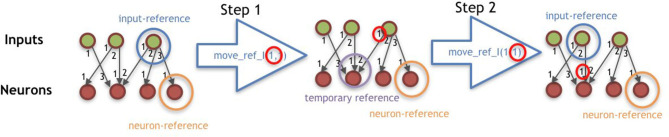
Illustration of command move_ref_I(*t*_*i*_, *d*_*i*_), having *t*_*i*_ = 1 and *d*_*i*_ = 1. The procedure of the command move_ref_N(*t*_*n*_, *d*_*n*_) is analogous to this.

From the beginning until the end of the string, each symbol is interpreted and developed. Nonetheless, for multiple reasons explained below, it is possible that a symbol ends up not being expressed in the phenotype. Furthermore, a maximum amount of *m* modules is allowed in a morphology, so that during late-development, after reaching this maximum, any upcoming modules are not expressed in the phenotype. The late-development of the phenotype for morphology and controller is depicted in the flowchart of Figure 5 (top), and detailed hereafter, where we reference parts of this flowchart through Roman numerals:

- ***I***: Because the first symbol of the string is always *C*, it is the first module to be added to the morphology, and the *module-reference* is updated with it. At this moment, the references of left, front, right, and back of the turtle are, respectively, left, up, right, and down (for a robot seen from top-down).
- ***II***: The interpretation of any Morphology-mounting command updates the *slot-reference* with the slot indicated by the command. If the *slot-reference* is not empty, it is overwritten, meaning that the command used for setting this previous slot into the reference is not expressed.
- ***III***: If the symbol is a module, it is coupled with the command in the *slot-reference* (if there is one).
- ***IV***: The addition of new modules requires both a Morphology-mounting command and a module. If the slot-reference is empty when interpreting a module, the module is not expressed in the phenotype, except for the *C* module, which is the very first module and needs no mounting command. When the *module-reference* is a joint, an attempt to attach it to the front slot is made, regardless of the mounting command. When the *module-reference* is the core-component, if its left, front, and right slots are occupied, an attempt to attach it to the back slot is made, regardless of the mounting command. If the mounting attempt is made to a slot that is occupied, the module is not expressed, while the command remains in the *slot-reference*. If the newly mounted module intersects an existing one during the development, both the new module and its associated network node (if there is one) are not expressed. After mounting a new module, the *module-reference* remains in the parent module, and the *slot-reference* is emptied.
- ***V***: The Morphology-moving commands update the *module-reference* according to the slot defined by the command. If the *module-reference* is a joint, any Morphology-moving command moves to the front slot.
- ***VI***: The Controller-moving commands update the *neuron-reference* or *input-reference* according to the steps defined by the command and is divided into two steps. The steps are illustrated by Figure 6.
- ***VII***: The Controller-changing commands apply changes to the *neuron-reference* and/or *input-reference*, or to the edge connecting them. Controller-changing commands act upon the input/neuron nodes at the top (latest) of the stack. If there are no input/neuron nodes yet (according to the requirements of the command), the command is not expressed. If a newly mounted module is a joint, a new neuron is created possessing a connection weight that is drawn from a random uniform distribution between −1 and 1, and this neuron becomes the *neuron-reference*. When a new neuron is created, this generates an edge between this neuron and the *input-reference*. If there is no input yet, the neuron is stacked (oldest neuron remains as *neuron-reference*). If there is a stack of inputs, the new neuron is connected to all of them; for the edges, the input on the top of the list uses the weight possessed by the neuron, while all the other inputs in the stack use their own weights; finally, the stack is partially emptied keeping only the latest neuron, which becomes the *neuron-reference*. If a newly mounted module is a sensor, a new input is created possessing a connection weight that is drawn from a random uniform distribution between −1 and 1, and this input becomes the *input-reference*. When a new input is created, this generates an edge between this input and the *neuron-reference*. If there is no neuron yet, the input is stacked (the oldest input remains as *input-reference*). If there is a stack of neurons, the new input is connected to all of them; for the edges, the neuron on the top of the list uses the weight possessed by the input, while all the other neurons in the stack use their own weights; finally, the stack is partially emptied keeping only the latest input, which becomes the *input-reference*. For every new edge created from an input to a neuron, the edge is attributed a serial ID within the neuron. Analogously, for every new edge created from a neuron to an input, the edge is attributed a serial ID within the input.

An example of late-development is illustrated in Figure 5 (bottom).

### 3.6. Initialization

**Figure 7:**
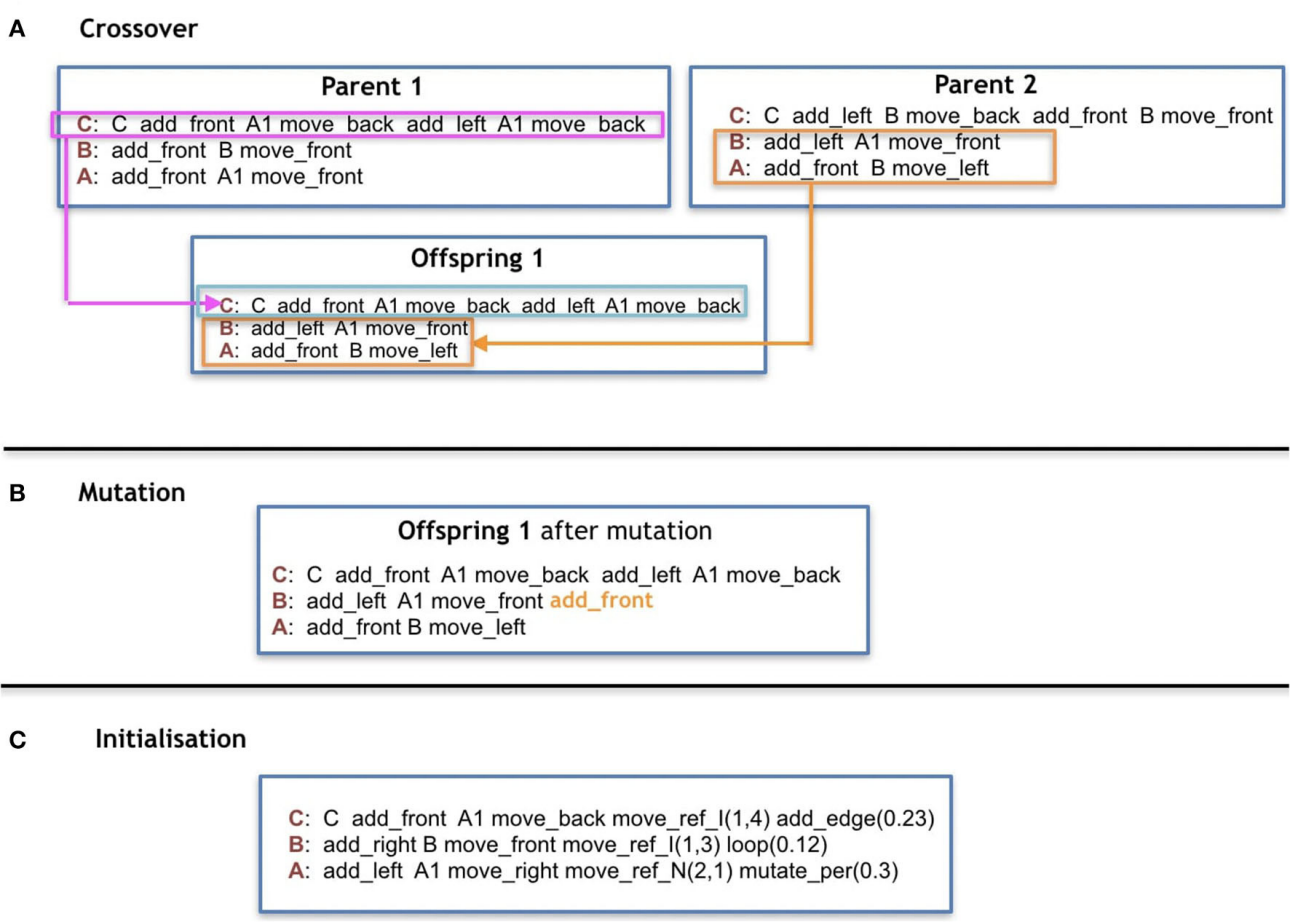
**(A,B)** Are examples of reproduction operators, and **(C)** is an example of initialization using only 1 group of symbols for all cases of rules.

To initialize a genotype in the *Baseline*, for each production-rule, exactly one symbol is drawn uniformly random from each of the following categories in this order: Controller-moving commands, Controller-Changing commands, Morphology-mounting commands, Modules, Morphology-moving commands. This process is repeated *s* times, being *s* sampled from a uniform random distribution ranging from 1 to *e*. This means that each rule can end up with 1 or maximally *e* sequential groups of five symbols. The symbol C is reserved to be added exclusively (and surely) at the beginning of the production rule C (Figure 7C).

In the case of the *Plasticoding*, the initialization of the production-rules is exactly the same as in *Baseline*, with the additional initialization of the regulatory clauses. Each regulation clause is initialized by selecting *z* random terms, each term selected from all the available environmental variables available. *z* can assume discrete values from 1 to *u*, each with equal probability, and the same term can be sampled multiple times. Each term is compared to a value chosen randomly between *True* or *False* with equal probability, and if *z* is above 1, the terms are connected by operator(s) chosen randomly between *and* or *or* with equal probability.

### 3.7. Crossover

For the *Baseline*, the crossovers are performed by selecting individual production-rules, each represented by one replaceable symbol. The selection is performed uniformly at random from the parents (Figure 7A).

In *Plasticoding*, the process is similar to *Baseline*, in the sense that groups of production-rules are selected individually, together with their regulatory clauses, where the grouping of production-rules is defined so that each group must be associated to the same replaceable symbol.

### 3.8. Mutation

There is an equal chance of a mutation happening to any production-rule of a grammar. For the production-rule chosen to be mutated, there is an equal chance of adding/deleting/swapping one random symbol from a random production-rule/position or apply a change to its regulation clause (Figure 7B). All symbols have the same chance of being removed or swapped. As for the addition of symbols, all categories have an equal chance of being chosen to provide a symbol, and every symbol of the category also has an equal chance of being chosen. An exception is made to *C* to ensure that a robot has one and only one core-component. This way, the symbol *C* cannot be added to any other production rules, neither removed nor moved from the production rule of *C*. The operations adding/deleting/swapping have an equal chance to happen. In the case of changing a regulation clause (for *Plasticoding*), there is an equal chance of adding/removing a term from the clause (by still ensuring that the number of terms is between [1, *u*], or flipping a variable of a term from *True* to *False* (or the inverse), or to flip an operator from *and* to *or* (or the inverse).

### 3.9. Evolution

**Figure 8:**
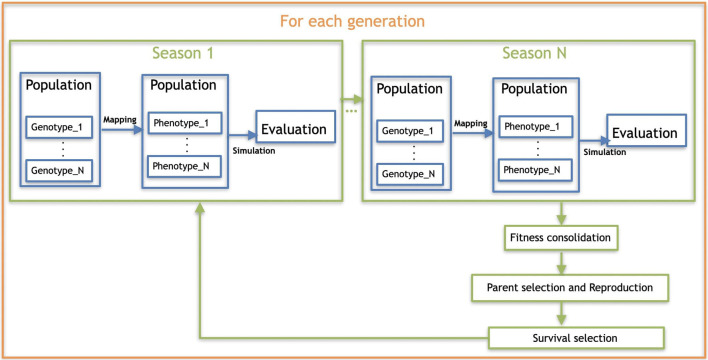
Overall scheme of the evolutionary process.

We are using overlapping generations with a population size μ = 100. In each generation, λ = 100 offspring are produced by selecting 100 pairs of parents through binary tournaments (with replacement) and creating one child per pair by crossover and mutation. From the resulting set of μ parents plus λ offspring, 100 individuals are selected for the next generation, also using binary tournaments. The evolutionary process is stopped after 200 generations, thus a total of 20,000 fitness evaluations per run are performed. The evaluation of each robot is done after the robot lives through each of the seasons, given that development takes place each time a season is started during a robot's life. Details about the seasonal environmental condition are provided in section 4.1, and an illustration of the whole evolutionary process is depicted by Figure 8. For each encoding, the experiment was repeated 20 times independently. A summary of the parameters for the evolutionary algorithm is provided in the list below:

- Population size 100
- Offspring size 100
- Number of generations 200
- Mutation probability 80%
- Crossover probability 80%
- Experiment repetitions 20
- Rewriting iterations k 3
- Maximum number of groups of symbols e 4
- Maximum number of terms per clause u 2
- Number of tuples per non-replaceable symbol l 2
- Connections of the network range from -1 to 1
- Oscillator parameters range from 1 to 10
- Maximum amount of modules m 15

## 4. Experimental Setup

We carried out two sets of experiments using the same experimental setup, except for the encoding method. The first set of experiments used the *Baseline* encoding, while the second set used the *Plasticoding* encoding. Our experiments were realized using a simulator called Gazebo, interfaced through a robot framework called Revolve (Hupkes et al., 2018). The code needed to reproduce our experiments and analysis in available on GitHub^3^, and the data is available (upon request) in the server ssh.data.vu.nl inside the karinemiras-frontiers2020 directory.

### 4.1. Environmental Conditions

**Figure 9:**
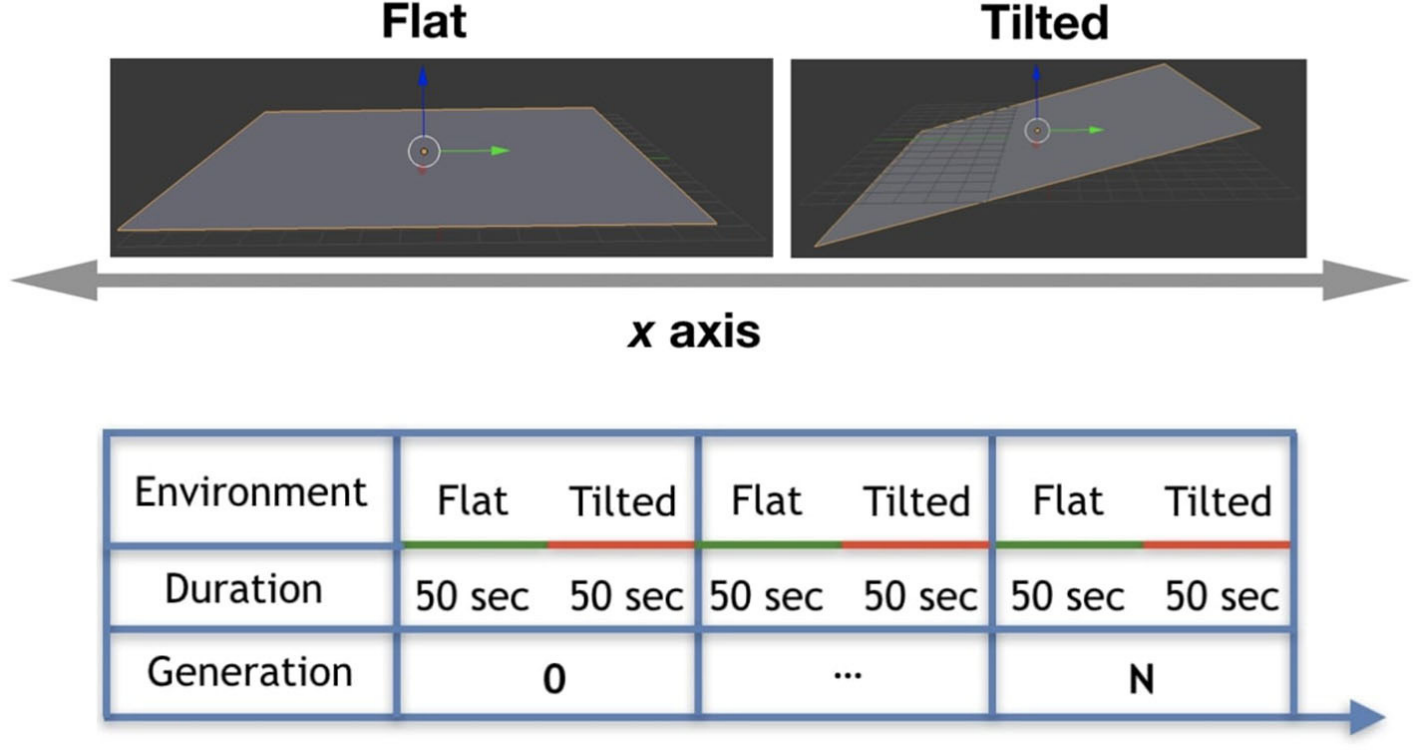
(Top) Environmental conditions: Flat and Tilted. (Bottom) Cycle of seasonal environmental condition.

We experimented with two different environmental conditions, which are (a) Flat environmental condition: it is a plane flat floor; (b) Tilted environmental condition: it is a plane floor tilted in 5°. These conditions are depicted in Figure 9 (top). In the experiments, these conditions were combined to create a seasonal environmental condition where each condition is considered a season. In practice, robots live each part of their lifetime in one different environmental condition. They spend their first 50 s of lifetime in the Flat environmental condition (first season), and after that they spend 50 more seconds in the Tilted environmental condition (second season), as depicted by Figure 9 (bottom). Note that during morphogenesis robots are in the Flat environmental condition, and later on during their life, have the chance to experience the Tilted environmental condition regardless their performance in the previous condition.

### 4.2. Fitness Function

For each environmental condition independently, Flat and Tilted, the fitness function measures performance on the task of directed locomotion. The function is defined by Equation (2).

(2) $$\begin{array}{l} f_{1}=\{\begin{array}{cc} s_{x} & \text{if }s_{x}>0\\ \frac{s_{x}}{10} & \text{if }s_{x}<0\\ -0.1 & \text{if }s_{x}=0 \end{array} \end{array}$$

where *s*_*x*_ is the speed of the robot as defined by Equation (4). This function measures the speed of the robots only in the *x* axis, so to discourage robots to exploit locomotion in the *y* axis, avoiding the proposed challenge of climbing the Tilted environmental condition. Additionally, there are two penalties to try to escape local optima observed in preliminary tests with the Tilted environmental condition. The first penalty is the division by 10 used when the speed is negative, which aims to prevent that a “safe strategy” be much more beneficial than completely falling down the hill. This “safe strategy” is characterized by trying to avoid falling too far from the starting point (due the effect of gravity), but without really climbing. The second penalty is the constant −0.1 used when speed is zero, which aims at disincentivizing robots that do not develop joints (and thus cannot move) so to avoid the risk of falling. Although these penalties are needed only in the Tilted environmental condition, they are used for the Flat condition as well, aiming to keep the experimental setup comparable. In any case, the penalties make sense for both environmental conditions because they increase the selection pressure for the task, i.e., directed locomotion.

Because robots are evaluated in multiple environmental conditions, we treat this problem as multi-objective, where the fitness of each environmental condition represents one of the objectives. Notably, this setup in which robots need to perform well in different environmental conditions can be seen as robots having to perform well different tasks. To obtain the final fitness, which represents the fitness in the seasonal environmental condition, we consolidate the two fitness values into a single measure. The consolidation of these objectives into the final fitness is defined by Equation (3).

(3) $$\begin{array}{l} f_{c}=\overset{n}{\underset{i=1}{\sum }}d_{i} \end{array}$$

where *d*_*i*_ is the number of solutions in the population that solution *i* dominates, given that a solution only dominates another solution if it is better in at least one objective and not worse in any objective.

### 4.3. Robot Descriptors

For quantitatively assessing morphological, control, and behavioral properties of the robots, we utilized a set of descriptors.

#### 4.3.1. Behavioral Descriptors

**1. Speed**: Describes the speed (cm/s) of the robot along the *x* axis as defined by Equation (4).

(4) $$\begin{array}{l} s_{x}=\frac{e_{x}-b_{x}}{t} \end{array}$$

where *b*_*x*_ is *x* coordinate of the robot's center of mass at the beginning of the simulation, *e*_*x*_ is *x* coordinate of the robot's center of mass at the end of the simulation, and *t* is the duration of the simulation.

**2. Balance**: We use the rotation of the head in the *x*–*y* plane to define the balance of the robot. In general, the rotation of an object can be described in the dimensions roll, pitch, and yaw. We consider the pitch and roll of the robot head, expressed in degrees between 0 and 180 (because we do not care if the rotation is clockwise or anti-clockwise). Perfect Balance belongs to both pitch and roll being equal to zero, so that the higher the Balance, the less rotated the head is. Formally, Balance is defined by Equation (5).

(5) $$\begin{array}{l} B=1-\frac{r+p}{t*180*2} \end{array}$$

where $r=\overset{t}{\underset{i=1}{\sum }}|r_{i}|$, representing the roll rotation accumulated over time, $p=\overset{t}{\underset{i=1}{\sum }}|p_{i}|$, representing the pitch rotation accumulated over time, and *t* is the duration of the simulation.

#### 4.3.2. Morphological Descriptors

**Figure 10:**
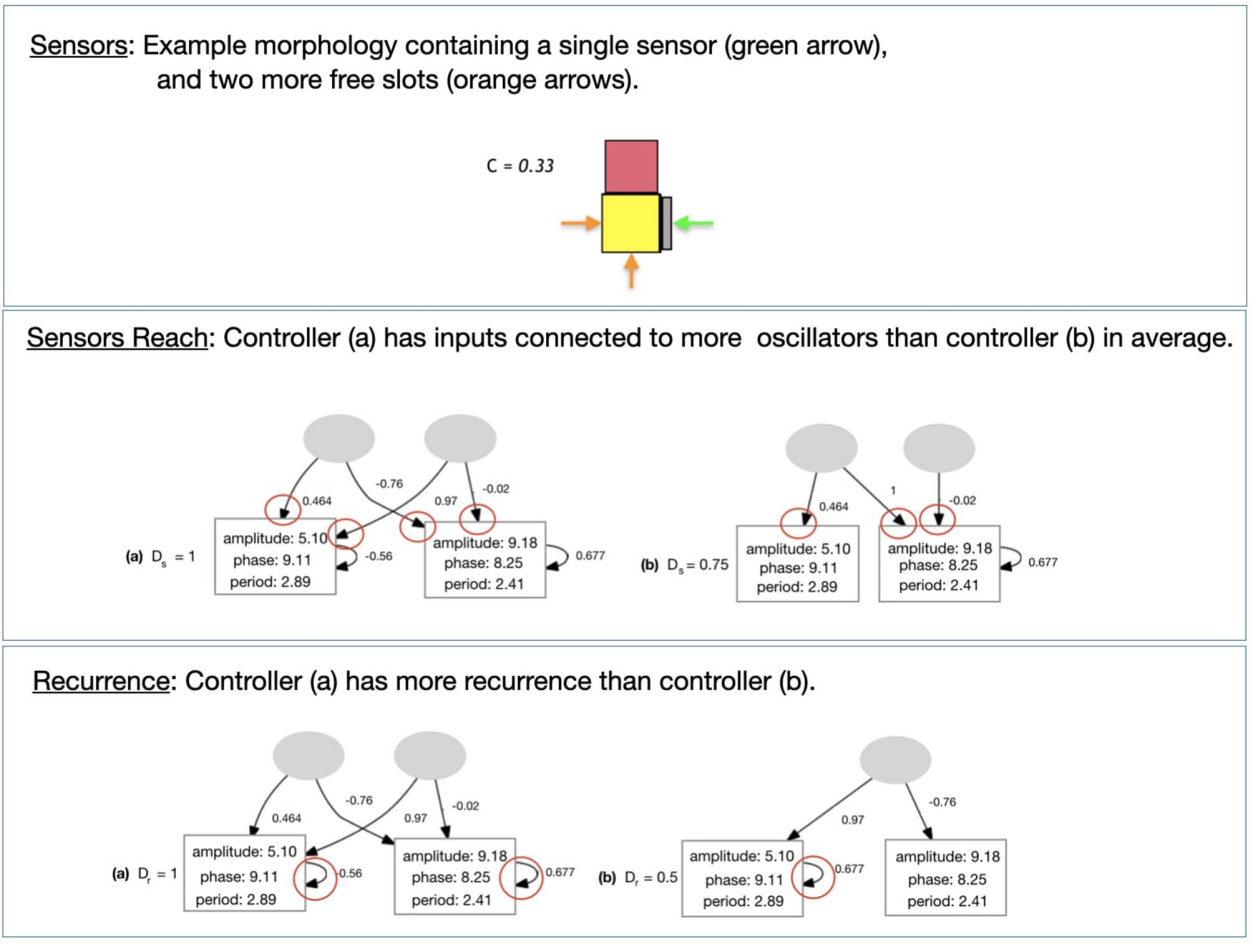
Illustrations for the descriptors calculations.

1. **Size**: Total number *S* of modules in the morphology.
2. **Sensors** Accounts for touch sensors in the morphology (Figure 10). It is defined with Equation (6):
  (6) $$\begin{array}{l} C=\{\begin{array}{cc} \frac{c}{c_{max}}, & \text{if }c_{max}>0\\ 0 & \text{otherwise} \end{array} \end{array}$$
  where *c* is the number of sensors and *c*_*max*_ is the number of slots in the morphology that are not connected to other types of module.

#### 4.3.3. Controller Descriptors

1. **Sensors Reach**: Describes how the inputs from the sensors are connected to the oscillators of the controller. The higher this number, the more motors each sensor is sending data to on average (Figure 10). It is defined with Equation (7):
  (7) $$\begin{array}{l} {\;D}_{s}=Md\left(R_{s}\right)\\ R_{s}=\{r_{s}|r_{s}=\frac{c_{s}}{n\left(L\right)}\forall s\in S\} \end{array}$$
  where *R*_*s*_ is a set of ratios, while *c*_*s*_ is the number of connections of the input *s*, *S* is the set of all inputs in the controller, and *n*(*L*) is the number of oscillators in the controller.
2. **Recurrence**: Describes the proportion of oscillators in the controller that have a recurrent connection, i.e., memory (Figure 10). It is defined with Equation (8):
  (8) $$\begin{array}{l} D_{r}=\frac{r\left(L\right)}{n\left(L\right)} \end{array}$$
  where *n*(*L*) is the number of oscillators of the controller, and *r*(*L*) is the number of oscillators that have a recurrent connection.

A complete search space analysis of the utilized robot framework and its descriptors is available in Miras et al. (2018a,b), demonstrating the capacity of these descriptors to capture relevant robot properties, and proving that this search space allows high levels of diversity.

## 5. Results and Discussion

**Figure 11:**
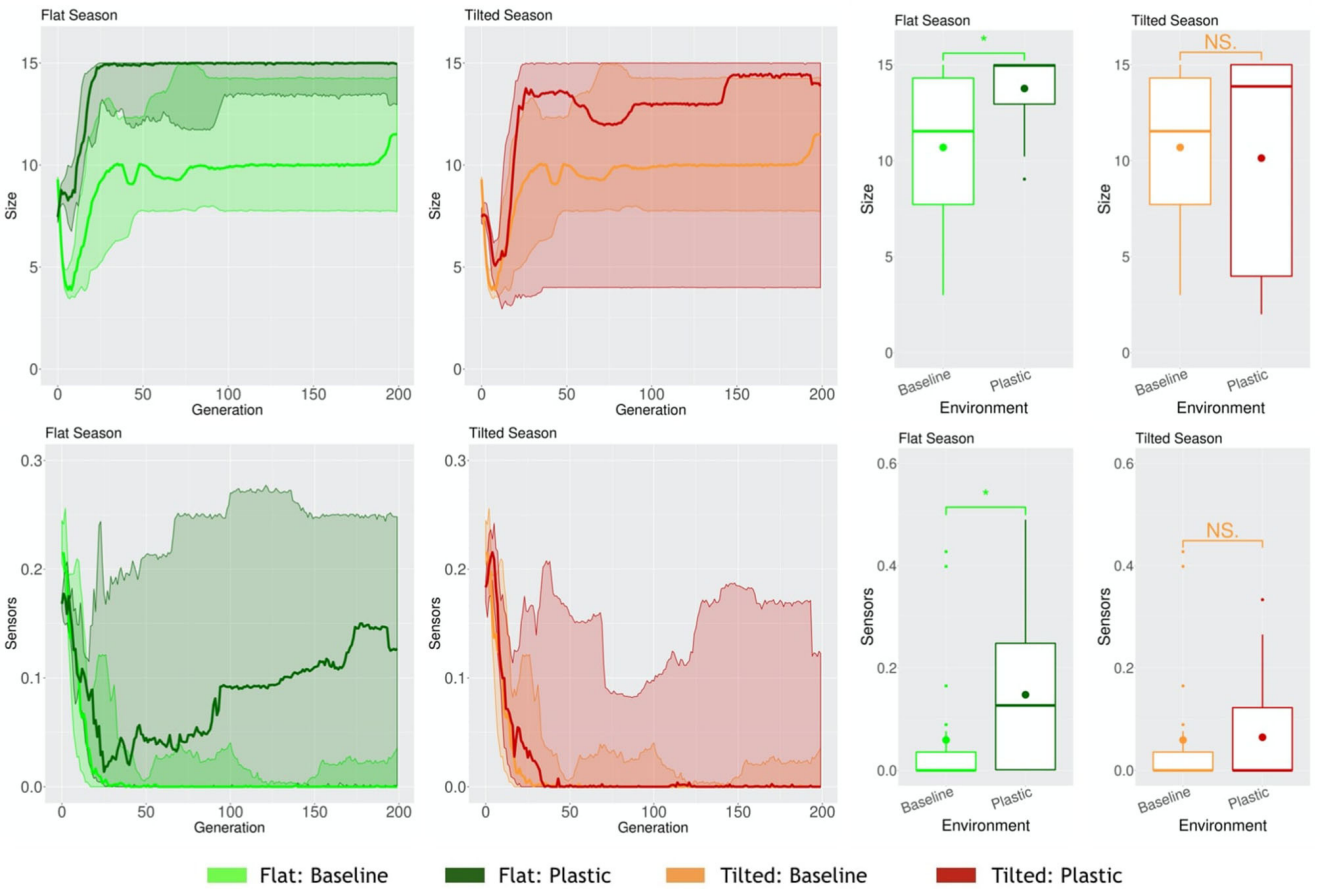
Comparison of morphological properties in different environmental conditions. Line plots show the progression of the means of the population (quartiles over all runs), while boxplots show the distribution of the means in the final generation. Significance levels for the Wilcoxon tests in the boxplots are * < 0.05, *NS* not significant. Note that, naturally, the values of Baseline are the same in Flat or Tilted.

**Figure 12:**
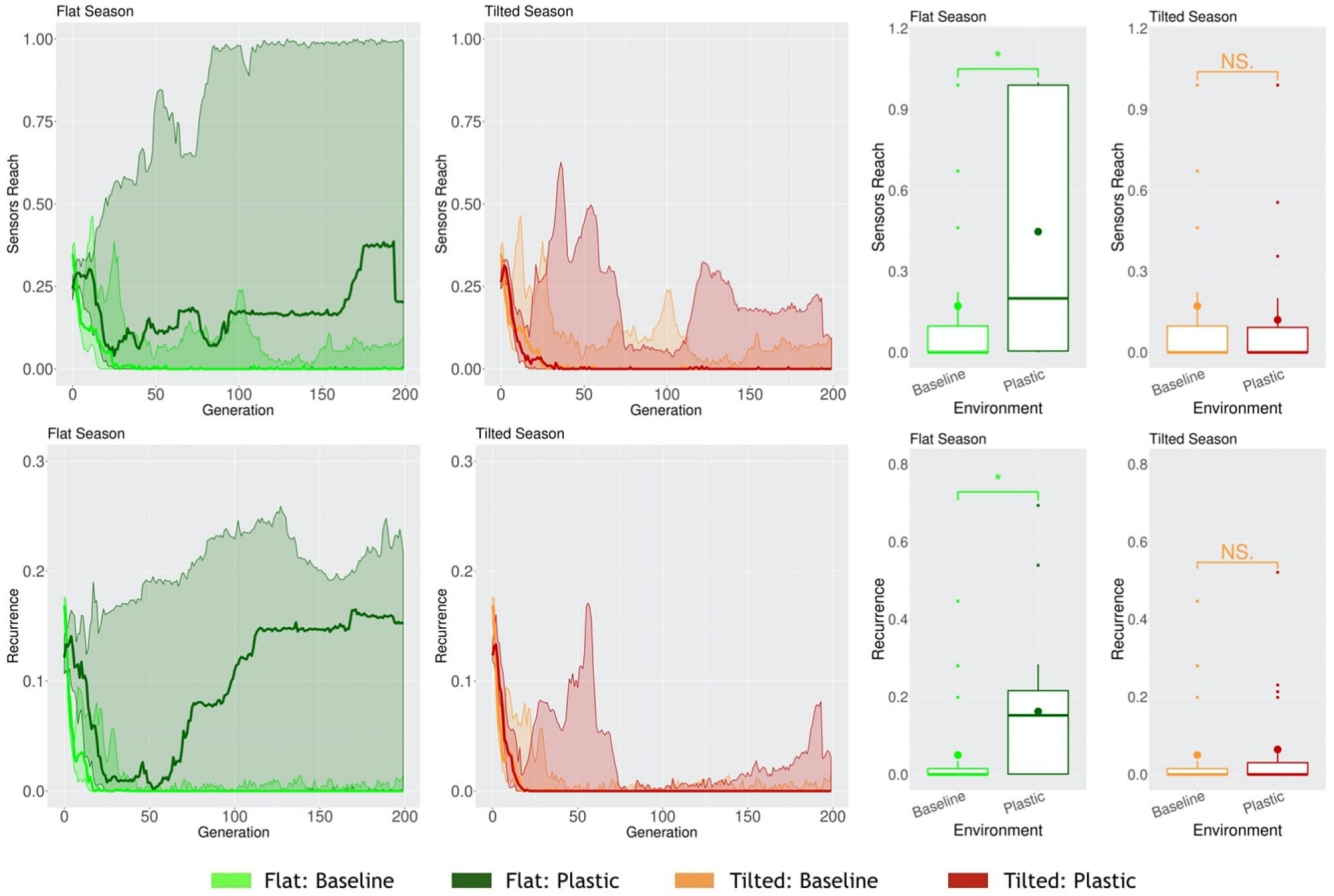
Comparison of controller properties in different environmental conditions. Line plots show the progression of the means of the population (quartiles over all runs), while boxplots show the distribution of the means in the final generation. Significance levels for the Wilcoxon tests in the boxplots are * < 0.05, *NS* not significant. Note that, naturally, the values of Baseline are the same in Flat or Tilted.

When robots have to cope with multiple environmental conditions while disposing of one same morphology and controller, and thus behavior, naturally, a trade-off may occur. Because of the need to adapt to different environmental conditions, in at least one of the environmental conditions they might adapt worse than if they had evolved in that same static environmental condition. We demonstrated this in a previous work (Miras et al., 2020b). In this case, for a seasonal environmental condition, where both Flat and Tilted had to be faced by the population, the Tilted season exerted a higher selection pressure. This way, robots acquired the same traits as robots that had evolved in a static Tilted environmental condition. One probable reason for this is that, as demonstrated in another study (Miras and Eiben, 2019b), robots evolved in an inclined environment can still perform the task in the a flat environment, but fail badly when it is the other way around, showing that the pressure of an inclined environment leads to more generalist strategies for locomotion. Because the new encoding that we propose in the current paper, i.e., *Plasticoding*, allows the same individual to develop distinct morphologies and/or controllers (and thus also behavior) according the environmental conditions, we expect the system to be less impacted by this trade-off. Therefore, here we compare two populations separately evolved in a seasonal environmental condition, (a) for one population the encoding method was *Baseline*, (b) for another population the encoding method was *Plasticoding*. The morphological properties, in the Flat season robots are bigger for *Plasticoding* than for *Baseline*, while they also have more sensors (Figure 11). Concerning controller properties, we observe differences that directly relate to sensor differences in morphology: The Flat season for the *Plasticoding* robots have higher Sensors Reach and Recurrence (Figure 12). This indicates that, in *Plasticoding*, robots evolve to have sensors sending signals to more motors, and that the brain of the robot has more memory, when compared to *Baseline*. Notably, given that the neurons of the controllers are oscillators, recurrence can only make a difference if there are inputs. In simpler words, memory is only needed if there is something to remember, and this may explain why both metrics (Sensor Reach and Recurrence) are increased at the same time. Therefore, the selection pressure for higher Recurrence in the Flat environmental condition suggests that sensors are useful in the context of seasons, provided that robots have capacity for *phenotypic plasticity*.

**Figure 13:**
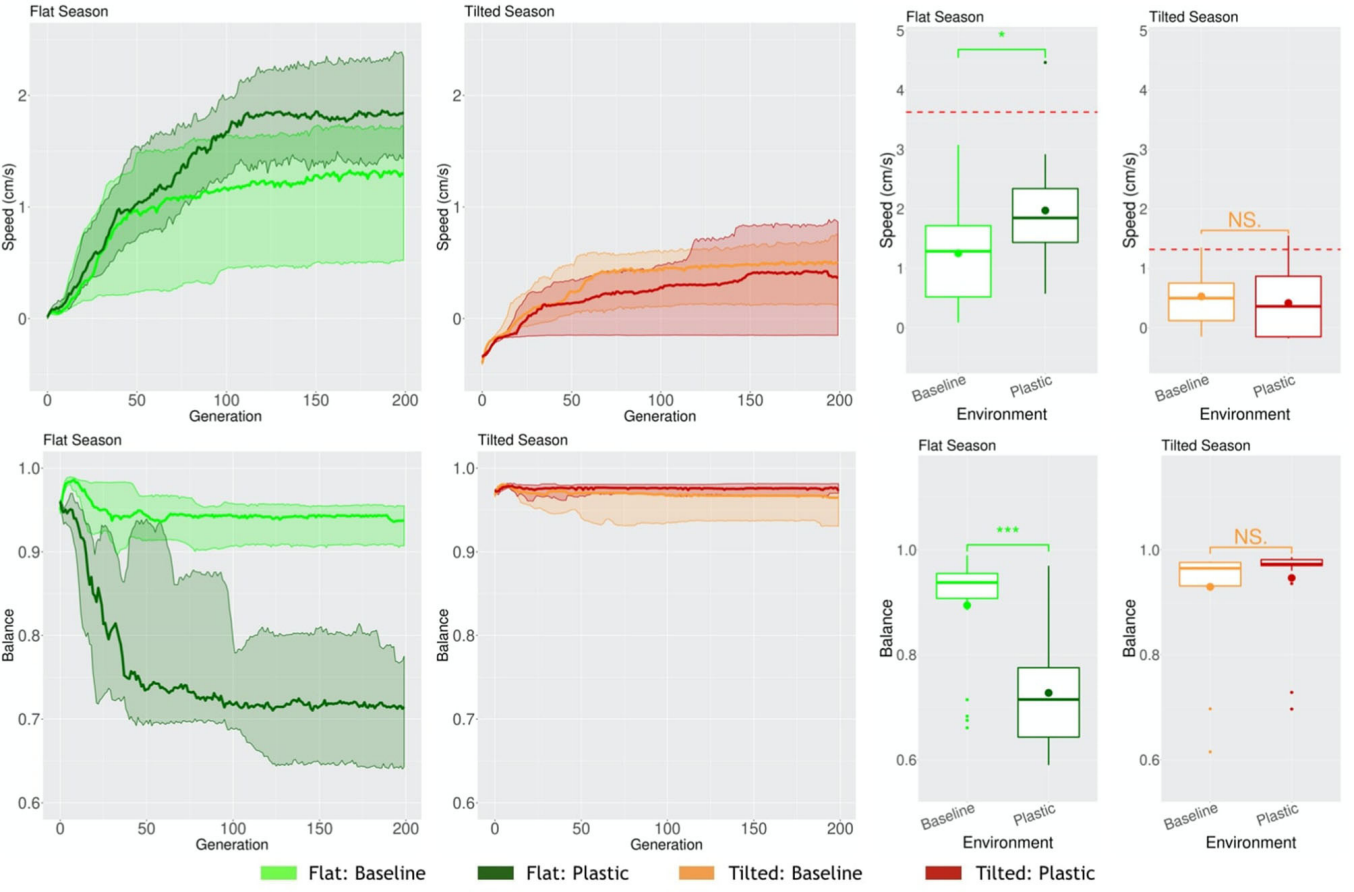
Comparison of behavioral properties in different environmental conditions. Line plots show the progression of the means of the population (quartiles over all runs), while boxplots show the distribution of the means in the final generation. Significance levels for the Wilcoxon tests in the boxplots are * < 0.05, * * * < 0.001, *NS* not significant. The red dotted lines represent the mean Speed when evolving in a static environmental condition.

In the Flat environmental condition, this phenotypic differentiation is clearly reflected on the emergent behavior, i.e., behavior that emerges from the interaction among morphology, controller, and environment to achieve the rewarded behavior (task). The Balance of the robots is lower for *Plasticoding* than for *Baseline* (Figure 13), and this behavioral property agrees with their predominant gait, which is rolling for *Plasticoding* and rowing or dragging for *Baseline*. While rolling requires an imbalance at the center of mass of the robots, rowing and dragging requires the opposite. Note that this rolling gait was expected to be observed because rolling is a common emergent behavior when evolving in a static Flat environmental condition (Miras and Eiben, 2019a). Nevertheless, though rolling is predominant for *Plasticoding* in Flat, this is not the case for *Baseline*, which predominantly delivers a gait of rowing/dragging, which is more common when evolving in a static Tilted environmental condition (Miras and Eiben, 2019a) instead. This corroborates with our discussion in the beginning of this section, concerning a pressure for the most generalist strategy for locomotion.

Finally, the rewarded behavior (task) shows that the phenotypic and behavioral changes caused by *Plasticoding* helped to improve the performance on the task when in the Flat season. The Speed of the evolved population is 58% higher for *Plasticoding* than for *Baseline*. This difference was proven to be significant with a Wilcoxon test presenting a *p*-value of 0.015 (Figure 13). It is no surprise that *Baseline* delivers robots that perform worse on the task when in the Flat environmental condition, considering that the *Baseline* gave in to the selection pressure existent in the Tilted environmental condition for robots that row and drag.

The red dotted lines in the boxplots of Speed (Figure 13) mark a reference for a “known achievement.” These lines represent the means of Speed when evolving populations in a static environmental condition, i.e., the environmental condition were always the same through the their lifetime, and serve as a reference of what could be achieved in a less constrained scenario. This leaves us with an open question: is it possible through *phenotypic plasticity* to achieve a performance non-different from when evolving in static environmental conditions, or is this degradation at least to some extent, inevitable given the costs of evolving regulatory capabilities?

**Figure 14:**
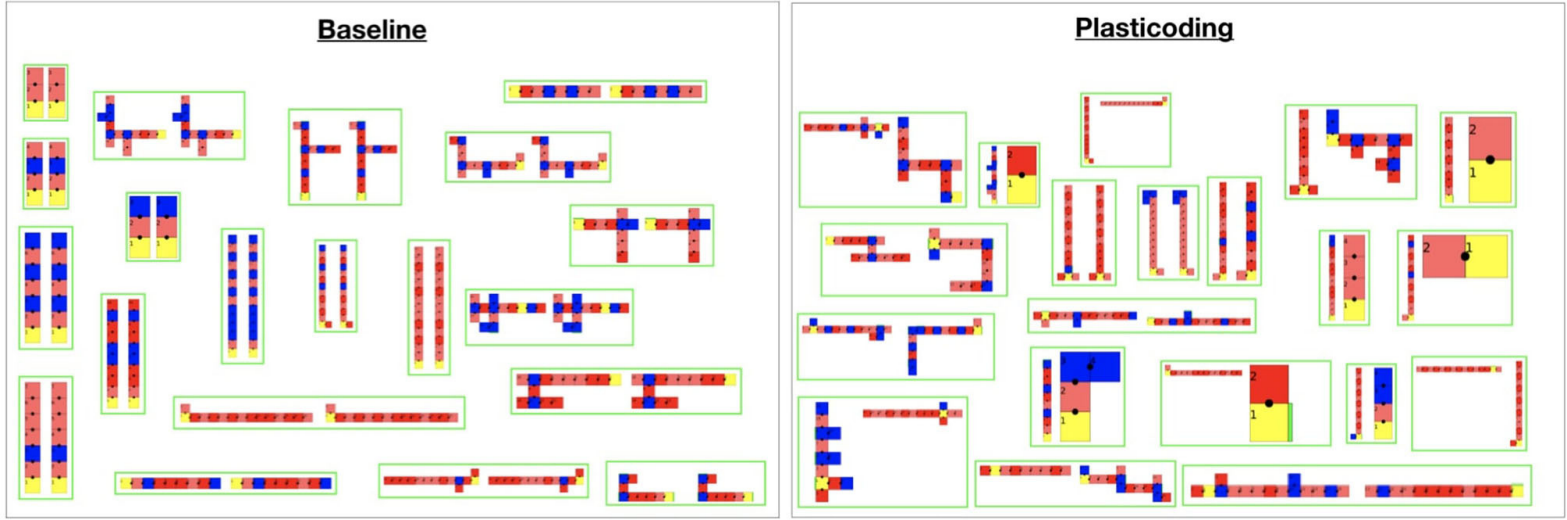
Illustrations of the best robots for each experiment repetition using each encoding method. At the left of each green box is the morphology developed in the Flat season, while at the right is the morphology developed in the Tilted season. Note that for the Baseline, naturally, in each green box both morphologies are the same. These are top-down 2D illustrations that use a polygon representing each module, assuming every module is the same size: each polygon color is relative to one type of module, as described earlier in Figure 1. Illustrations were rescaled to fit the frames accordingly. A video showing examples of best emergent robots is available on the link https://www.youtube.com/watch?v=43wsQfWMo-Q&feature=youtu.be.

Importantly, all aforementioned differentiation between *Plasticoding* and *Baseline* that took place in the Flat season did not take place in the Tilted season. This is also true for the task performance, for which no gain or loss was achieved. One possible explanation might be the fact that the Tilted environmental condition is more challenging (Miras and Eiben, 2019a) than the Flat. In Figure 11, by observing the curves of Size, we see that until around generation 25 the search is trying to escape the local optimum mentioned in section 4.2. That is, first the population turns into very small robots, then later on they grow bigger. Notwithstanding, although we see a stable increase in the average, there is a lot of variance maintained until the end on the evolutionary period. Figure 14 helps to illustrate that, showing that it is common to end up with very small robots, so small that they can barely locomote. Perhaps one explanation to this is that the obvious difficulty of evolving robots in the Tilted environmental condition led evolution to exploiting the Flat environmental condition instead.

## 6. Concluding Remarks

We investigated the effects of environmental regulation on the evolution of robots using a novel encoding method that we called *Plasticoding*. This regulation gave robots a capacity for *phenotypic plasticity*, so that one same robot could develop a different morphology, controller, or behavior given changes in environmental conditions. In a set of experiments, we evolved robots that had to cope with two different environmental conditions during their life: one flat floor and one inclined floor. Importantly, each of these conditions presents a different selection pressure (Miras and Eiben, 2019b). This means that in each one of these environments, the most likely emergent morphological and behavioral properties are significantly different. By comparing the results achieved by *Plasticoding* to a baseline encoding (similar encoding but with no regulation capacity), we showed that environmental regulation improves robot adaptation while leading to different evolved morphologies, controllers, and behavior.

The novel encoding method that we proposed and utilized is designed for a particular system of robot modules. However, this paper presented a proof of concept concerning the benefits of *phenotypic plasticity* through environmental regulation that transcends the encoding utilized for the concept demonstration. Furthermore, the method of regulation adopted by *Plasticoding* is independent of the robot system and applicable to any simple L-System because it disregards the content of the production rules. The environment is determinant to natural life forms not only indirectly through creating selection pressure, but also directly through acting upon development (Sapolsky, 2017). For these reasons, we believe environmental regulation has great potential in helping to improve the quality of ER systems. Nevertheless, this subject is very scarcely explored in the literature. Therefore, our work is a fundamental step toward a long-term vision: succeeding in creating robot artificial life with complexity and adaptability comparable to what we see in nature. For future work we propose to improve *Plasticoding* through experimenting with a) the mutation probabilities, trying to balance changes in the production-rules vs. regulatory clauses; b) different methods of initialization for the production-rules and regulatory clauses. Additionally, we propose to investigate effects on evolvability through a) limiting *phenotypic plasticity* to occur during morphogenesis only; b) allowing the inheritance of regulatory changes (*epigenetics*). Finally, the effects of environmental regulation should be investigated using additional environmental conditions, and these conditions should occur through diverse dynamics of change, e.g., fast changes, slow changes, cyclical changes, etc.

## Data Availability Statement

The datasets generated for this study are available on request to the corresponding author.

## Author Contributions

KM formulated the hypothesis, designed and implemented the system, performed the analysis, and wrote most of the text. EF helped to analyze the results and wrote part of the text. AE helped with the design choices of the system, helped to analyze the results, and wrote part of the text. All authors contributed to the article and approved the submitted version.

## Conflict of Interest

The authors declare that the research was conducted in the absence of any commercial or financial relationships that could be construed as a potential conflict of interest.

## Footnotes

^1^Another part of the DNA is often denoted as “junk,” that is, it has either no function or (most likely) it has a function that we do not know of yet.

^2^This example shows how future extensions with multiple environmental variables may look like.

^3^https://github.com/ci-group/revolve/tree/ae99a0985765997e5e5b557bc677f4cc1bc84c99/experiments/plasticoding_frontiers2020
